# Supplementary figures and images for: Descriptive profile of risk factors for cardiovascular diseases using WHO STEP wise approach in Madhya Pradesh
Source: PeerJ. 2020 Aug 6;8:e9568. doi: 10.7717/peerj.9568 (PMC7415222; doi:10.7717/peerj.9568)

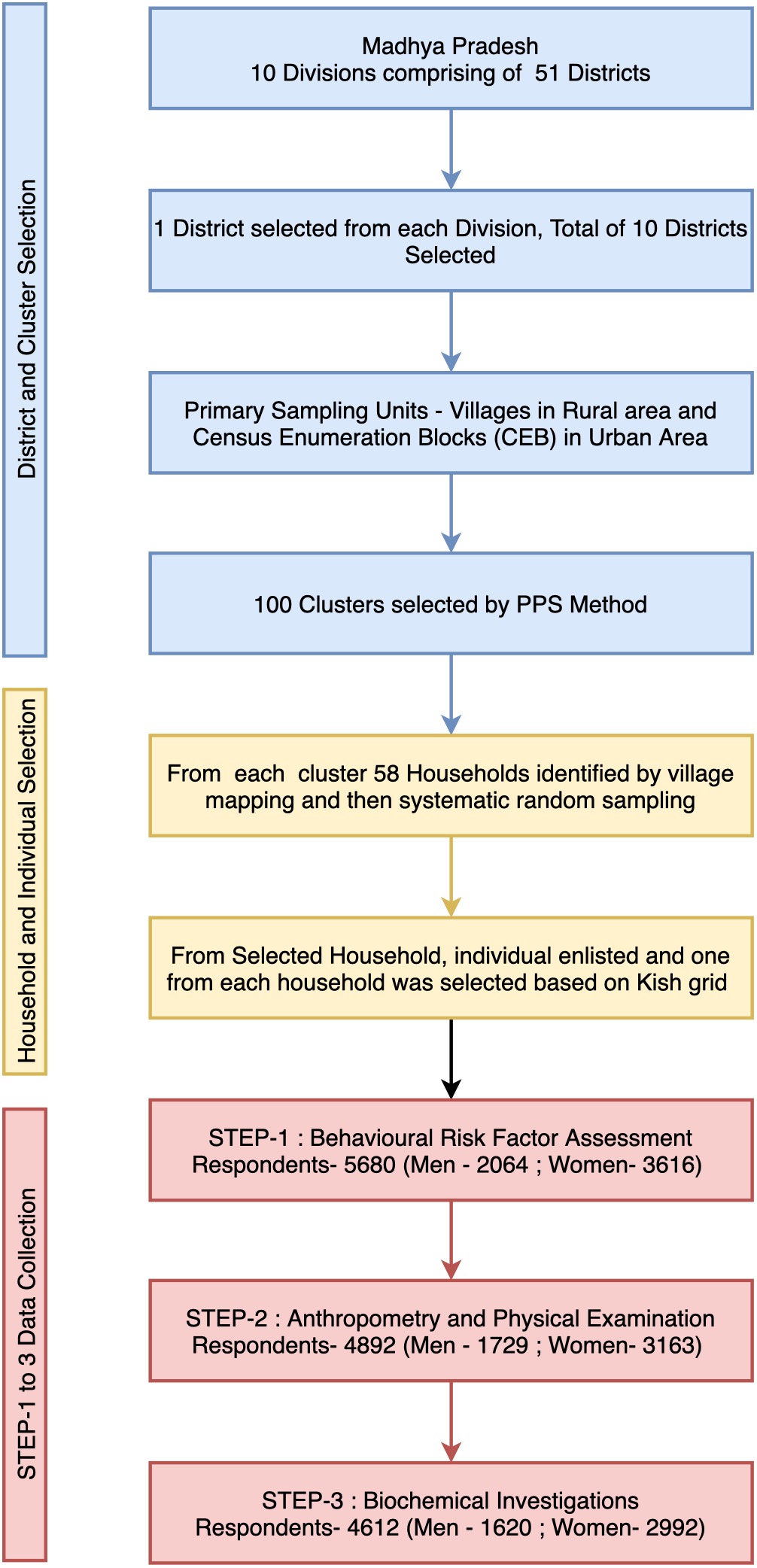

Supplement: Supplemental Information 2 [file peerj-08-9568-s002.jpg]
